# From Pathology to Formulation: Designing Biodegradable Polymers for Personalized Drug Delivery

**DOI:** 10.3390/pharmaceutics18030330

**Published:** 2026-03-06

**Authors:** Mariann Dinya, Elek Dinya, Gábor M. Mórotz

**Affiliations:** 1 Department of Pharmacology and Pharmacotherapy, Faculty of Medicine, Semmelweis University, H-1089 Budapest, Hungary; 2 Center for Pharmacology and Drug Research & Development, Semmelweis University, H-1089 Budapest, Hungary; 3 Institute of Digital Health Sciences, Semmelweis University, H-1089 Budapest, Hungary; dinya.elek@semmelweis.hu

**Keywords:** biodegradable polymers, pathological microenvironment, disease-driven design

## Abstract

**Highlights:**

**What are the main findings?**

- Analysis of 65 in vivo studies reveal disease-specific polymer–trigger patterns
- Pathological microenvironments guide rational polymer carrier design.

**What are the implications of the main findings?**

- Ionizable polysaccharide and methacrylate systems dominate intestinal inflammation.
- Enzyme- and redox-responsive polymers align with joint and tumor diseases.
- Multi-responsive carriers improve robustness in heterogeneous environments.

**Abstract:**

**Background/Objectives:** Selection of polymer carriers for targeted drug delivery is typically guided by material availability or trigger responsiveness rather than disease-specific evidence. However, successful preclinical formulations may already encode implicit design rules linking polymer composition to particular pathological environments. This study aimed to identify reproducible material-disease associations across biodegradable polymer systems and to derive formulation-oriented guidance for disease-calibrated carrier selection. **Methods:** A structured synthesis of 65 preclinical in vivo studies (2020–2025) covering inflammatory bowel disease, arthritis, cardiovascular inflammation, and solid tumors was performed. Extracted variables included polymer family, backbone chemistry, stimulus responsiveness, disease model, and reported therapeutic benefit relative to controls. Associations between polymer composition, trigger mechanisms, and disease categories were analyzed using cross-tabulation, chi-square statistics, Cramér’s V, and direction-of-effect synthesis. **Results:** Distinct material-disease clustering patterns emerged. Ionizable polysaccharide and methacrylate systems (e.g., alginate, chitosan, Eudragit) were strongly associated with intestinal inflammatory models, reflecting reliance on pH- and ion-mediated mechanisms. Enzyme-degradable hyaluronic acid matrices were concentrated in joint and cartilage disorders characterized by protease overexpression. Oxidation-sensitive polyether systems (e.g., PEG-PPS) and redox-active hybrid platforms predominated in atherosclerosis and tumor models, where oxidative stress is a defining pathological feature. Composite and multi-responsive systems were disproportionately represented in tumors, consistent with microenvironmental heterogeneity. Across studies, therapeutic improvement was consistently reported when polymer functional motifs aligned with dominant biochemical drivers of the disease. **Conclusions:** Successful biodegradable polymer carriers exhibit disease-specific compatibility patterns rather than universal applicability. These recurring associations suggest that polymer selection can be guided by pathological context even in the absence of direct outcome comparisons. The resulting formulation-oriented framework supports rational carrier choice for personalized drug delivery based on disease-specific microenvironment signatures.

## 1. Introduction

Modern pharmaceutical manufacturing, particularly in generic drug development, relies on a relatively small repertoire of well-characterized polymer excipients selected primarily for safety, scalability, regulatory acceptance, and manufacturing feasibility. Although these materials enable robust and reproducible formulations, they are frequently applied across diverse therapeutic areas without explicit consideration of disease-specific microenvironmental conditions [1,2,3,4,5,6,7,8,9,10,11,12,13]. Consequently, material selection for targeted therapies often remains largely empirical, despite evidence that pathological tissues exhibit markedly altered biochemical environments, including changes in pH, redox balance, enzymatic activity, and ionic composition. Targeted drug-delivery systems aim to enhance therapeutic efficacy while minimizing systemic toxicity by concentrating pharmacological activity at diseased sites. Biodegradable polymer carriers have become central to this strategy because they allow controlled drug release through diffusion, matrix degradation, and stimulus-responsive mechanisms. In particular, stimuli-responsive systems exploit endogenous pathological cues such as extracellular acidity, oxidative stress, protease activity, or inflammation-associated biochemical changes to achieve site-specific activation. pH-responsive methacrylate polymers and polysaccharide matrices have enabled localized intestinal drug delivery, while redox-responsive platforms target oxidative stress across inflammatory, ischemic, and neoplastic tissues [14,15,16,17,18,19]. Enzyme-responsive matrices further extend this concept by coupling drug release to disease-associated protease activity. Matrix metalloproteinase-responsive hydrogels, for example, have been developed for both osteoarthritis and inflammatory bowel disease, where elevated protease levels correlate with tissue destruction and inflammation [20,21]. Such systems demonstrate that pathological microenvironments can serve as functional triggers for drug delivery rather than passive barriers. 

Different diseases present distinct microenvironmental signatures, motivating the development of tailored carrier systems. Inflammatory bowel disease has driven extensive research into pH-sensitive, microbiota-responsive, and mucoadhesive platforms capable of distal intestinal targeting [3,4,5,6,7,12]. Cardiovascular inflammation and atherosclerosis have been addressed using reactive oxygen species (ROS)-responsive nanoplatforms, including shear-sensitive designs that respond to oxidative stress and altered hemodynamics within plaques [18,22,23]. Protease-rich joint environments have been targeted using enzyme-degradable hydrogels and hyaluronic-acid-based matrices designed for sustained intra-articular release [20,21,24]. In oncology, multifunctional carriers combining pH, redox, or metal-ion sensitivity seek to address tumor heterogeneity and dynamic microenvironmental conditions [25,26,27]. Despite rapid progress, most formulation strategies are developed within individual disease models and interpreted in isolation. As a result, knowledge embedded in successful systems, such as recurring material choices for specific pathological environments, remains fragmented across the literature. Polymer classes exhibit characteristic physicochemical behaviors that may confer advantages in particular microenvironments, yet whether these theoretical compatibilities translate into reproducible patterns of material selection across studies has not been systematically examined.

Direct comparison of therapeutic efficacy across preclinical models is complicated by heterogeneity in animal species, disease induction methods, outcome measures, and dosing regimens. Nevertheless, the consistent observation of therapeutic benefit relative to controls suggests that the literature collectively represents a repository of experimentally validated design solutions. Analyzing this landscape may reveal implicit formulation principles guiding the development of disease-targeted carriers.

The present study synthesizes evidence from 65 preclinical in vivo investigations published between 2020 and 2025 across inflammatory bowel disease, arthritis and cartilage disorders, cardiovascular inflammation, and solid tumors. Rather than comparing efficacy across heterogeneous models, we examined associations between polymer composition, stimulus-responsive mechanisms, and disease context to identify recurring material-disease pairings. Our objectives were to determine which classes of biodegradable polymers are most frequently associated with specific pathological conditions, to assess whether single- or multi-responsive systems predominate in different environments, and to derive formulation-oriented guidance that may support rational carrier selection for context-dependent and potentially personalized therapeutic strategies.

## 2. Materials and Methods

### 2.1. Search Strategy, Data Sources, and Eligibility Criteria

A systematic literature search was conducted in the PubMed and Scopus databases to identify preclinical in vivo studies published between 1 January 2020 and 31 August 2025.

PubMed and Scopus were selected because together they provide comprehensive coverage of biomedical, pharmacological, and materials science literature relevant to stimulus-responsive polymer drug-delivery systems, with substantial overlap with other major indexing databases.

The search strategy combined controlled vocabulary and free-text terms addressing three main domains: biodegradable polymeric drug delivery systems, stimulus responsiveness (including ion-, pH-, ROS-, enzyme-, or dual-responsive mechanisms), and animal models of inflammatory or neoplastic diseases. The full Boolean search strings for each database are provided below to ensure reproducibility.

The search was performed independently by two reviewers (M.D. and G.M.M.) using the same predefined strategy in duplicate rounds across both databases to maximize retrieval completeness and minimize selection bias. Inter-reviewer agreement during screening was assessed using Cohen’s kappa coefficient, and disagreements were resolved by discussion or consultation with a third reviewer (E.D.).

**Figure 1:**
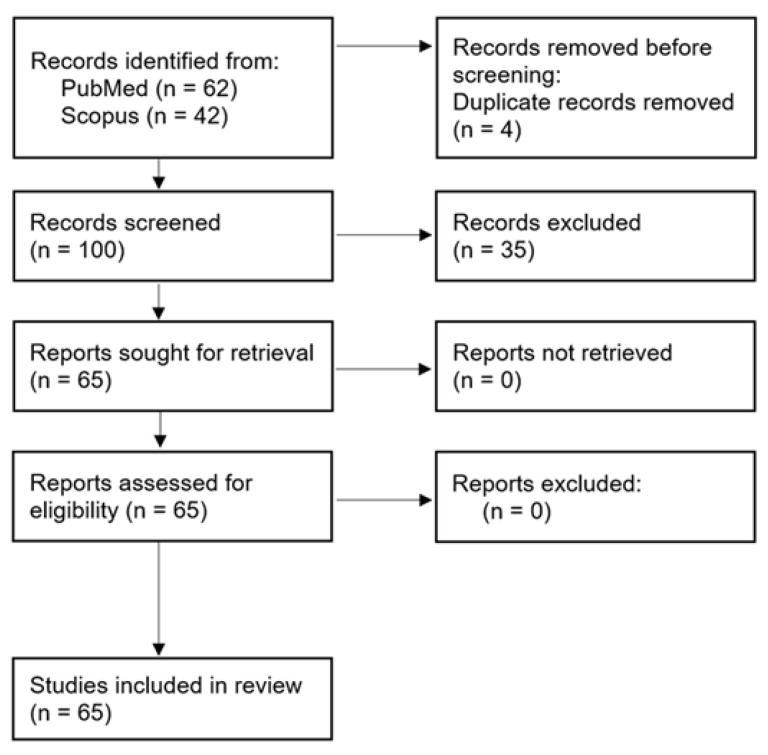
PRISMA flow diagram of study selection. Database searches were conducted in PubMed and Scopus databases. Identification yielded 104 records (PubMed: 8 + 54; Scopus: 27 + 15). After removing 4 duplicates, 100 records were screened at the title/abstract level, and 35 were excluded. In total, 65 studies were included in the qualitative synthesis. Reasons for exclusion are summarized in the Methods section.

The database search yielded 104 records (PubMed n = 62; Scopus n = 42). After removal of four duplicate entries, 100 records remained for title and abstract screening, of which 35 were excluded as clearly irrelevant. Sixty-five full-text articles met the predefined eligibility criteria and were included in the final analysis (Figure 1). This systematic review followed the PRISMA 2020 guidelines [28].

PUBMED search query:

(“polymer matrix”[tiab] OR “polymeric matrix”[tiab] OR hydrogel[tiab] OR microsphere*[tiab] OR nanoparticle*[tiab])

AND (“drug delivery”[tiab] OR “controlled release”[tiab] OR “sustained release”[tiab] OR “modified release”[tiab] OR “drug release”[tiab])

AND ((blood[tiab] OR serum[tiab] OR plasma[tiab])

AND (“ion environment”[tiab] OR “ionic environment”[tiab] OR “ionic microenvironment”[tiab] OR “electrolyte”[tiab] OR “ionic strength”[tiab] OR sodium[tiab] OR potassium[tiab] OR calcium[tiab] OR magnesium[tiab] OR phosphate[tiab])

AND (inflammation[tiab] OR colitis[tiab] OR arthritis[tiab] OR atherosclerosis[tiab] OR sepsis[tiab] OR hepatitis[tiab] OR pancreatitis[tiab])

AND (in vivo[tiab] OR animal*[tiab] OR mouse[tiab] OR rat[tiab] OR rabbit[tiab] OR murine[tiab]) NOT (review[pt] OR “systematic review”[tiab] OR “meta-analysis”[tiab] OR case reports[pt] OR ocular[tiab] OR ophthalmic[tiab] OR wound[tiab] OR transdermal[tiab]) AND (“1 January 2020”[Date—Publication] : “31 August 2025”[Date—Publication])

SCOPUS search query:

TITLE(“polymer matrix” OR “polymeric matrix” OR hydrogel OR microsphere OR nanoparticle) AND TITLE-ABS-KEY(“drug delivery” OR “controlled release” OR “sustained release” OR “modified release” OR “drug release”) AND TITLE-ABS-KEY((blood OR serum OR plasma) AND (human OR patient* OR clinical OR volunteer* OR cohort OR mouse OR mice OR murine OR rat OR rats OR rabbit*)) AND TITLE-ABS-KEY((“ionic strength” OR electrolyte* OR sodium OR potassium OR calcium OR magnesium OR phosphate OR “ion environment” OR “ionic environment” OR “ionic microenvironment” OR “electrolyte environment” OR “ionic milieu” OR “electrolyte milieu”) W/5 (release OR “drug release” OR “controlled release”)) AND TITLE-ABS-KEY(inflammation OR inflammatory OR colitis OR arthritis OR “inflammatory bowel disease” OR atherosclerosis OR sepsis OR hepatitis OR pancreatitis OR cancer OR tumor OR carcinoma OR neoplasm*)AND PUBYEAR > 2019 AND PUBYEAR < 2026AND LIMIT-TO(DOCTYPE, “ar”)AND LIMIT-TO(LANGUAGE, “English”)

Eligibility criteria were defined according to the Population-Intervention-Comparator-Outcome (PICO) framework (Table 1). Studies were included if they investigated biodegradable polymer-based carriers with stimulus-responsive drug release in preclinical in vivo models of inflammatory or neoplastic disease, regardless of delivery route, and were reported as original research articles. Studies were excluded if they were reviews, meta-analyses, or conference abstracts; used non-biodegradable or non-polymeric carriers; focused exclusively on wound healing, ocular, or transdermal applications; or reported only in vitro data without in vivo validation. Articles not meeting the inclusion criteria for the primary analysis could nevertheless be cited elsewhere for contextual purposes.

The search strategy was intentionally designed to capture studies investigating microenvironment-responsive polymer systems across inflammatory and neoplastic conditions, including those influenced by ionic, pH, redox, or enzymatic factors, without restricting the search to specific polymer classes or delivery platforms.

### 2.2. Data Extraction

Data were independently extracted from all eligible studies by the same two reviewers described above using a standardized spreadsheet developed specifically for this analysis. Extracted variables included bibliographic information (first author, publication year, journal), disease model characteristics (species, induction method, pathological subtype), polymer type and formulation features, primary stimulus responsiveness (pH, ion, ROS/redox, enzyme, dual/multi-responsive, or other), route of administration, therapeutic endpoints (e.g., tumor volume, histological score, inflammatory markers, oxidative stress indicators, survival), and quantitative outcome measures where available (means, standard deviations, standard errors, *p*-values, or confidence intervals).

Discrepancies were resolved by discussion and, when necessary, adjudication within the author team. All numerical data were extracted directly from the text or tables of the original publications; graphical digitization was not performed. Because of substantial heterogeneity across disease models, polymer chemistries, stimulus mechanisms, and reported endpoints, quantitative synthesis was feasible only for subsets of studies with sufficient comparability.

To capture broader structure-function relationships across heterogeneous experimental designs, a complementary descriptive framework was applied. This included vote counting and direction-of-effect assessment to summarize outcome patterns across polymer families, trigger types, and disease categories. Given that most included studies represented proof-of-concept investigations reporting therapeutic improvement relative to controls, outcomes were classified primarily according to the presence or absence of reported benefit. For mapping analyses, formulations demonstrating biologically or statistically meaningful improvement over control conditions were categorized as positive outcomes, whereas neutral or negative findings were recorded only when explicitly reported. This approach enabled consistent evaluation of associations between material design parameters and therapeutic performance rather than direct comparison of efficacy across fundamentally different models.

#### Classification of Stimulus Responsiveness

Stimulus responsiveness was classified according to the primary release-driving mechanism explicitly described in each full-text article. Each formulation was assigned to one predefined category based on the dominant mechanism governing in vivo drug activation: pH-responsive, ROS/redox-responsive, enzyme-responsive, ion-responsive, dual or multi-responsive, or other mechanisms.

pH-responsive systems included formulations activated by protonation-deprotonation processes, pH-dependent solubility transitions, acid-labile linkages, or enteric dissolution behavior. ROS-responsive systems comprised carriers destabilized by oxidative stress through redox-labile bonds or oxidation-induced structural changes. Enzyme-responsive systems required enzymatic cleavage or degradation mediated by disease-associated enzymes such as matrix metalloproteinases, hyaluronidase, or microbial enzymes. Ion-responsive systems were classified as such only when ion exchange or ion-dependent crosslink dissociation was explicitly identified as the primary trigger of drug release.

Formulations exhibiting multiple responsiveness (e.g., pH+ROS, pH+ion, ROS+enzyme) were categorized as dual or multi-responsive when at least two stimuli contributed to activation. Constituent stimulus components were additionally recorded to enable secondary mapping analyses of trigger combinations. Intrinsic ionic effects inherent to polymer structure (e.g., polyelectrolyte swelling or charge screening) were not considered independent triggers unless explicitly identified by the original authors as the principal release mechanism. Systems driven primarily by targeting ligands, biomimetic coatings, physical cues, or other non-canonical mechanisms were assigned to the “other” category.

Classification decisions were based on the descriptions provided in the original studies and were applied consistently across all included formulations.

Detailed characteristics of the included studies and extracted parameters are presented in Supplementary Table S1. All included studies are listed and referenced in Supplementary Table S1 [14,15,16,17,18,19,20,21,22,23,24,25,26,27,29,30,31,32,33,34,35,36,37,38,39,40,41,42,43,44,45,46,47,48,49,50,51,52,53,54,55,56,57,58,59,60,61,62,63,64,65,66,67,68,69,70,71,72,73,74,75,76,77,78,79].

### 2.3. Risk of Bias Assessment

Risk of bias in individual studies was evaluated using a modified version of the SYRCLE tool for animal experiments [3]. The assessment covered key methodological domains including randomization procedures, allocation concealment, blinding of investigators and outcome assessors, sample size reporting, completeness of outcome data, and selective reporting.

Given the exploratory nature and substantial heterogeneity of the included preclinical studies, the bias assessment was used primarily to identify methodological limitations and potential sources of uncertainty rather than to exclude studies from the analysis.

### 2.4. Statistical Analysis

Where quantitative outcome data were sufficiently comparable, effect sizes were calculated as standardized mean differences (Hedges’ g) with 95% confidence intervals using a random-effects model (DerSimonian–Laird method).

Between-study heterogeneity was assessed using Cochran’s Q statistic, degrees of freedom (df), the H statistic (H = √(Q/df)), I^2^ (I^2^ = max{0,(Q − df)/Q} × 100%), and τ^2^. Approximate thresholds of 25%, 50%, and 75% were interpreted as low, moderate, and high heterogeneity, respectively [4].

#### 2.4.1. Effect Sizes, Subgroups, and Sensitivity

Data extraction included polymer type, stimulus-responsiveness category, disease model, route of administration, and primary therapeutic outcomes as reported by the original authors.

Pre-specified subgroup analyses examined formulation type (nanoparticle, hydrogel, microsphere), stimulus-responsiveness category, disease group (inflammatory vs. tumor), and route of administration (oral vs. parenteral). Sensitivity analyses included leave-one-out procedures and Baujat influence plots. Publication bias and small-study effects were evaluated using contour-enhanced funnel plots and Egger’s regression. Where asymmetry was suggested (*p* < 0.10), trim-and-fill analyses were performed.

#### 2.4.2. Sparse Data and Association Analyses

This hybrid analytical approach was adopted to accommodate the heterogeneity of preclinical models and the frequent absence of extractable quantitative parameters required for conventional meta-analysis.

Because many studies did not report extractable quantitative parameters, additional analyses were performed to identify structural relationships within the dataset. Vote counting and direction-of-effect plots were used to summarize outcome trends across studies. Associations between polymer families, trigger mechanisms, and disease categories were analyzed using chi-square tests, Cramér’s V, and standardized residuals.

#### 2.4.3. Overall Associations and Analytical Considerations

Across the 65 included studies, global associations between trigger type, polymer family, and disease category were evaluated using chi-square statistics and Cramér’s V. Standardized residual analyses were performed to identify localized over-representations within contingency tables. Vote counting was used to estimate the proportion of studies reporting positive, neutral, or negative outcomes across disease groups and trigger categories. Because most studies were proof-of-concept investigations reporting therapeutic improvement relative to controls, this approach enabled consistent comparison of outcome patterns across heterogeneous models.

Although a random-effects meta-analysis was prespecified, calculation of pooled effect sizes was not feasible for the majority of studies due to insufficient numerical reporting, with many outcomes presented only graphically. Therefore, association analyses and descriptive synthesis were prioritized, while the meta-analytic framework was retained for potential future updates if extractable data become available.

## 3. Results

### 3.1. Study Selection and Characteristics

To analyze the most recent advances in microenvironment-responsive drug delivery, our systematic search across PubMed and Scopus focused on studies published between 2020 and 2025. The query identified 104 records. After the removal of four duplicates, 100 articles were screened by title and abstract. Full texts of potentially eligible articles were retrieved and assessed for compliance with the inclusion criteria. Thirty-five studies were excluded due to ocular delivery, wound-healing indications, non-biodegradable materials, or absence of in vivo validation. Ultimately, 65 studies met the inclusion criteria and were included in the final synthesis (Figure 1).

The selected studies covered a wide spectrum of inflammatory and tumor-related disease models, including IBD, arthritis and cartilage disorders, atherosclerosis, hepatitis, pancreatitis, and solid tumors. Both murine and larger animal models (mouse, rat, rabbit) were employed, using standard induction methods such as DSS-induced colitis, collagen-induced arthritis, high-fat diet-induced atherosclerosis, and xenograft implantation. Across these models, formulations were based on biodegradable polymer systems with diverse backbone chemistries, including methacrylate copolymers (e.g., Eudragit), polysaccharides (chitosan, alginate, and hyaluronic acid), aliphatic polyesters (PLGA), gelatin-based matrices, PEG-derived block copolymers, and hybrid biomimetic constructs.

Many platforms combined natural and synthetic components, membrane coatings, or inorganic modules to achieve disease-specific responsiveness. Stimulus sensitivity encompassed pH, ion, ROS, and enzyme triggers, as well as dual- or multi-responsive systems, reflecting attempts to match carrier behavior to pathological microenvironments. Administration routes were predominantly oral and parenteral (intravenous, intraperitoneal, intratumoral).

In cardiovascular diseases, particularly in atherosclerosis, multiple studies focused on ROS-responsive platforms. ROS-responsive nanoassemblies enhanced macrophage targeting and attenuated atherosclerotic plaque progression [18,19,27,37,62], while cell-membrane-based and shear-stress-responsive systems promoted endothelial repair and plaque stabilization in ApoE^−^/^−^ models [22,60,62]. In acute myocardial infarction, inflammation-modulating nanoparticle systems reduced infarct size and myocardial fibrosis by suppressing inflammatory pathways and macrophage activation [53,79]. Microenvironment-responsive hydrogels have also been engineered for cardiovascular implants and tissue substitutes, where glycocalyx-mimetic or polymer-layered coatings improved antithrombosis, immunoregulation, and resistance to calcification [61,70].

In joint and cartilage disorders, including rheumatoid arthritis, osteoarthritis, and intervertebral disk degeneration disease, polymeric hydrogels responsive to matrix metalloproteinases (MMPs) and hyaluronic-acid-based composites were frequently applied. These systems enabled enzyme-triggered degradation and targeted intra-articular drug release, supporting localized therapy with reduced systemic exposure across multiple in vivo arthritis and cartilage repair models [7,20,24,34,49,54,55,58]. 

Tumor models, such as hepatocellular carcinoma, colorectal cancer, and breast cancer, highlighted the versatility of dual-responsive carriers. In colorectal cancer, ROS- and enzyme-sensitive nanoparticles improved drug penetration and tumor inhibition [35]. For hepatocellular carcinoma, polymer-based targeted nanocarriers provided selective release in the tumor microenvironment while reducing systemic toxicity [36,71].

Some excluded studies still provided contextual insights, such as applications in wound healing, ocular delivery, or non-biodegradable polymer systems [8,9,10,11].

### 3.2. Quantitative Synthesis and Mapping

The analyses in Section 3.2 describe patterns of application across studies (frequencies and associations) rather than comparative therapeutic efficacy.

#### 3.2.1. Trigger Type Distribution

**Figure 2:**
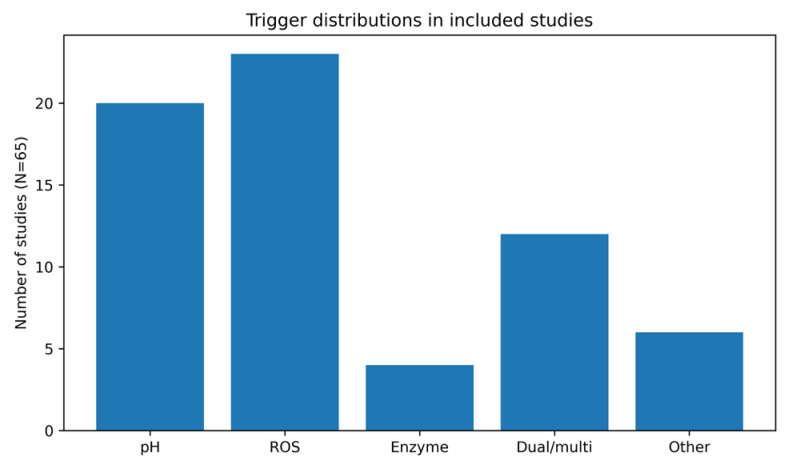
Trigger distribution across the 65 analyzed studies. ROS, reactive oxygen species.

First, we quantified the distribution of stimulus-responsive mechanisms across the 65 included studies using the harmonized trigger classification described in Section 2.2. ROS-responsive systems were reported in 23 studies (35.4%), pH-responsive platforms in 20 studies (30.8%), and dual- or multi-responsive formulations in 12 studies (18.5%). Approaches categorized as “other”, including ligand-mediated or mechanically responsive strategies, were reported in 6 studies (9.2%), while enzyme-responsive carriers appeared in 4 studies (6.2%) (Figure 2).

#### 3.2.2. Polymer Family Trigger Type Mapping

**Figure 3:**
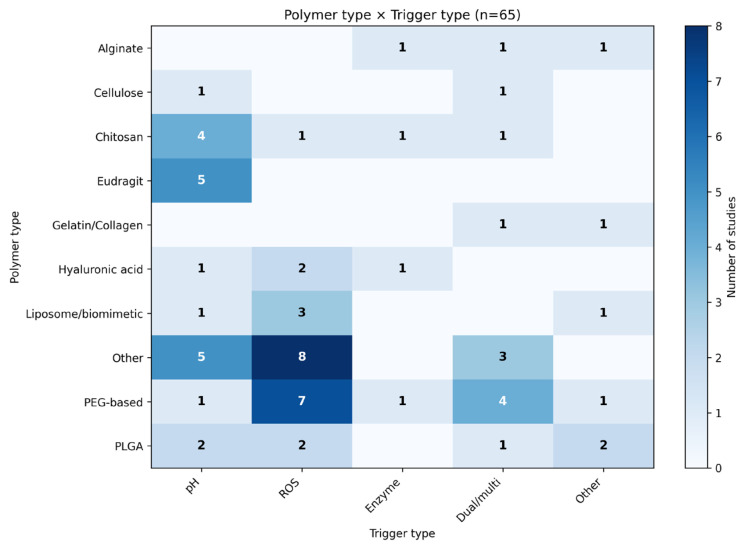
Polymer type-trigger type mapping across the 65 analyzed studies based on harmonized classifications. The heatmap shows the frequency of polymer classes (rows) associated with different stimuli-responsiveness (columns). Darker shading indicates higher study counts. PEG-based, poly(ethylene glycol)-derived copolymers; PLGA, poly(lactic-co-glycolic acid); ROS, reactive oxygen species.

Cross-tabulation of polymer families and stimulus types (Figure 3) revealed non-uniform responsiveness patterns across the included studies. Eudragit-based systems were pH-responsive (8 studies, 12.3%). Chitosan- and alginate-based carriers appeared primarily in pH-responsive categories and were also represented in ROS-responsive and multi-trigger formulations. PEG-PPS copolymers clustered within ROS-activated systems (4 studies, 6.2%). Hyaluronic acid (HA) platforms were represented mainly in ROS-related and dual-responsive designs (3 studies, 4.6%). PLGA-based carriers showed distribution across pH-, ROS-, and multi-responsive strategies (6 studies, 9.2%). MOF- and nanozyme-containing systems were linked mainly to ROS-dependent or combined triggers (3 studies, 4.6%).

Taken together, Figure 3 shows that polymer families were not evenly distributed across trigger categories, with some materials appearing predominantly in single-trigger designs and others recurring across multiple trigger types.

#### 3.2.3. Cross-Tabulation Analysis of Polymer Chemistry and Trigger Type

**Figure 4:**
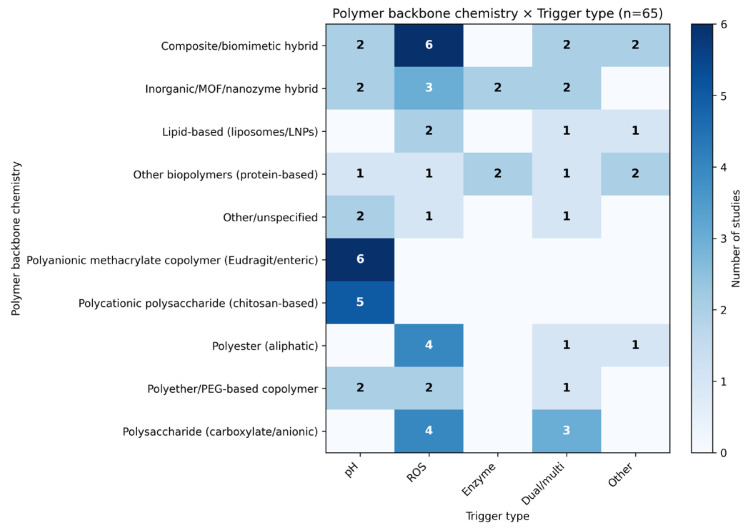
Cross-tabulation of polymer backbone chemistry and trigger type across the 65 included studies. The heatmap shows the frequency of associations between polymer chemistry classes (rows) and stimulus-responsive mechanisms (columns). Numbers indicate the number of studies for each combination, and darker shading corresponds to higher study counts. Dual/multi categories represent formulations responsive to more than one stimulus (e.g., pH+ROS, pH+ion, ROS+enzyme). MOF, metal–organic framework; ROS, reactive oxygen species.

To further resolve the patterns observed at the polymer family level, stimulus distribution was analyzed according to polymer backbone chemistry (Figure 4). Composite or biomimetic hybrid systems were represented across ROS-responsive and multi-trigger designs. Polyanionic methacrylate copolymers (e.g., Eudragit) appeared predominantly in pH-responsive studies, while polycationic polysaccharides (mainly chitosan-based systems) were likewise dominated by pH-driven release. Anionic polysaccharides were more frequently represented in multi-responsive designs. Aliphatic polyesters (PLGA/PLA) were distributed across ROS-responsive and combined-trigger categories. Polyethers, including PEG-derived copolymers such as PEG-PPS, clustered within ROS-activated systems.

Inorganic or catalytic materials, including MOF- and nanozyme-containing systems, were concentrated in ROS-related and combined categories. Crosslinked polymer networks appeared mainly in pH- and enzyme-responsive systems, consistent with cleavage- or degradation-controlled release. Protein-based matrices were rare and showed no dominant trigger profile.

Overall, Figure 4 shows that stimulus responsiveness follows polymer backbone chemistry rather than carrier form. Polyelectrolytes align with pH-dependent mechanisms, oxidation-sensitive polyethers with ROS triggers, degradable polyesters support broader strategies, and hybrid systems enable multi-trigger designs.

#### 3.2.4. Trigger Distribution Across Disease Groups

**Figure 5:**
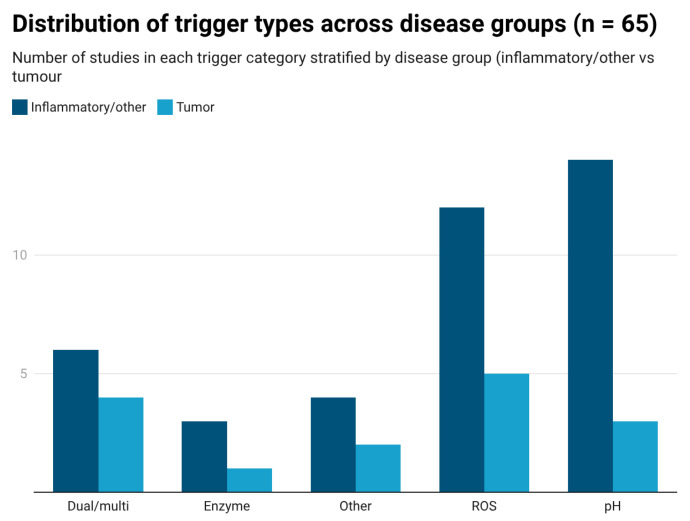
Distribution of trigger types across disease groups in the 65 included studies. Bars represent the number of studies employing each trigger mechanism in inflammatory versus tumor models. ROS, reactive oxygen species.

We analyzed the distribution of trigger types across disease groups to identify disease-specific design patterns (Figure 5). Inflammatory models, including colitis, arthritis, atherosclerosis, hepatitis, and pancreatitis, were predominantly associated with pH-responsive systems. ROS-responsive platforms were also frequently applied in inflammatory diseases, particularly in atherosclerosis models. Ion-responsive systems and enzyme-sensitive carriers were less frequent but were primarily confined to inflammatory contexts, especially gastrointestinal and joint disorders.

In contrast, tumor models were dominated by ROS-responsive and dual-responsive designs. Enzyme-responsive systems appeared only sporadically in tumor settings, whereas ion-associated mechanisms were rare outside inflammatory conditions.

Overall, trigger profiles differed between inflammatory and tumor models, with pH-associated mechanisms recurring more frequently in inflammatory conditions and ROS-based or multi-responsive systems recurring more frequently in tumor-targeted formulations (Figure 5).

#### 3.2.5. Polymer Chemistry Distribution Across Disease Groups

**Figure 6:**
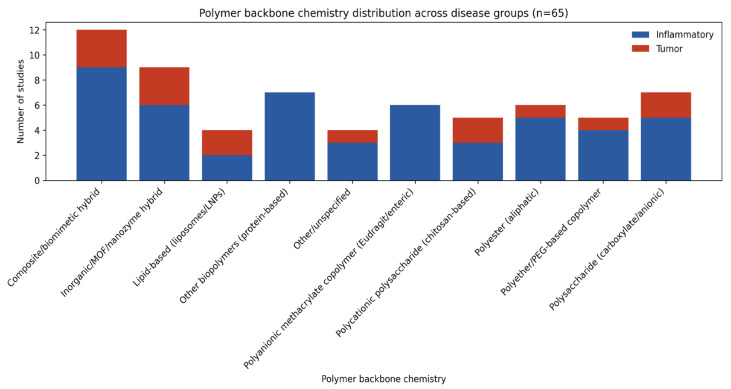
Polymer chemistry distribution across disease groups across 65 included studies. The stacked bar chart illustrates the number of studies focusing on inflammatory diseases (blue) or solid tumors (red) in relation with polymer chemistry. MOF, metal–organic frameworks.

Polymer backbone chemistry showed a clear disease-dependent distribution across the included studies (Figure 6). Composite and biomimetic hybrid systems constituted the largest category overall and were strongly enriched in inflammatory models. Polyanionic methacrylate copolymers (e.g., Eudragit) and protein-based matrices were likewise almost exclusively associated with inflammatory diseases.

In contrast, inorganic and MOF/nanozyme hybrid systems displayed a comparatively higher representation in tumor models, distinguishing them from most organic polymer classes. Aliphatic polyesters, polyethers, polysaccharide-based carriers, and lipid systems were present in both disease groups.

Other or unspecified systems were rare and showed no consistent disease preference. Overall, these data indicate that polymer selection is not uniform across indications but shows distinct distributions between inflammatory versus tumor-targeted applications (Figure 6).

**Figure 7:**
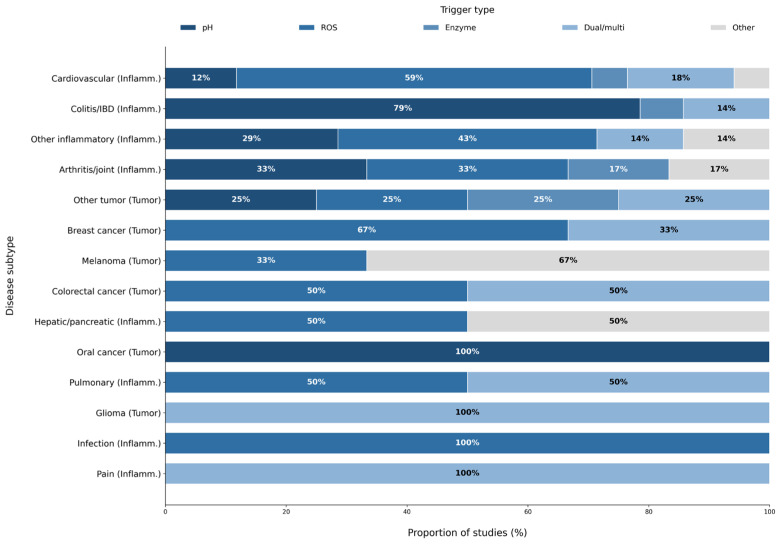
Normalized trigger-type distribution across inflammatory and tumor disease subtypes (n = 65). Stacked bars represent the percentage contribution of pH-, ROS-, enzyme-, dual/multi-, and other stimulus-responsive systems within each subtype. Normalization enables comparison of trigger selection patterns across disease contexts with unequal sample sizes.

To clarify how stimulus selection relates to specific pathological environments, trigger distributions were analyzed across disease subtypes (Figure 7). Distinct patterns emerged across disease subtypes. Across inflammatory conditions, pH- and ROS-responsive systems predominated, but their relative contributions differed substantially between subtypes.

Cardiovascular inflammatory models were dominated by ROS-responsive systems. These studies [19,27,37,41,42,60,62,64,75,79] primarily employed redox-responsive polymers or hybrid carrier systems. 

Colitis and inflammatory bowel disease models showed a strong bias toward pH-responsive platforms. Protonation-dependent behavior of ionizable polymers enabled localized drug release within the inflamed colon. Arthritis and joint disorders displayed a broader trigger profile. pH-responsive systems were represented by studies [24,34], ROS-responsive systems by [7,61], enzyme-responsive platforms by [20], and alternative trigger strategies by [58], indicating heterogeneous trigger usage across joint disease models. Other inflammatory conditions exhibited mixed trigger usage across studies.

Tumor models showed fewer studies overall but demonstrated clear subtype-specific tendencies. Breast cancer models (n = 3) were restricted to oxidative and dual-responsive designs, with ROS-responsive systems represented by [25,32] and dual/multi-responsive platforms by [23]. Melanoma models were associated primarily with non-classical or composite triggers; for example, study [52] utilized a pathogen-mimicking platform categorized as “Other”.

Colorectal cancer models relied on both ROS-responsive and dual-responsive carriers, represented by studies [26,35], respectively. Oral cancer models included pH-responsive systems such as [38].

Across inflammatory and tumor subtypes, trigger categories showed subtype-dependent distributions, with pH-, ROS-, enzyme-, and dual/multi-responsive systems recurring at different frequencies (Figure 7).

#### 3.2.6. Polymer Type-Trigger-Disease Interaction Network

**Figure 8:**
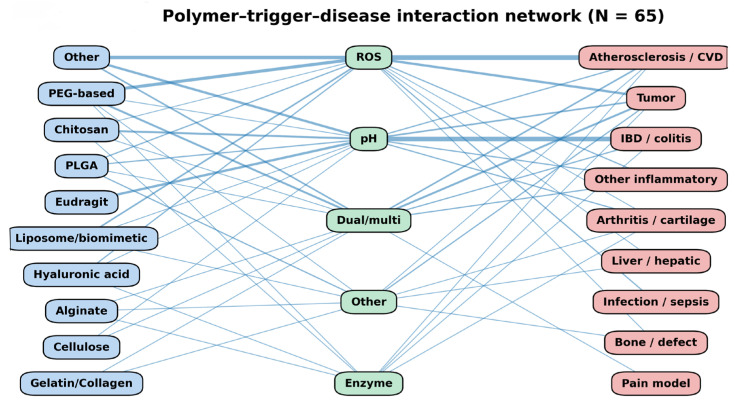
Polymer-trigger-disease interaction network. The tripartite network illustrates the relationships between biodegradable polymer families (blue nodes), dominant trigger mechanisms (green nodes), and associated disease model groups (red nodes) across 65 included preclinical studies (n = 65). Edge thickness corresponds to the number of studies supporting each association. The map highlights dominant formulation strategies linking polymer chemistry, microenvironmental responsiveness, and disease context.

Finally, we integrated the relationships between polymer families, dominant stimulus-responsiveness categories, and associated disease models into a tripartite network (Figure 8). The resulting network revealed distinct clusters among the analyzed groups.

Polyanionic methacrylate copolymers (Eudragit), together with chitosan- and alginate-based systems, were predominantly associated with pH- and ion-responsive triggers and were mainly linked to inflammatory bowel disease models. PEG-PPS and MOF/nanozyme-based systems formed a separate cluster around ROS-responsive triggers and were strongly associated with tumor and atherosclerosis models. HA-based hydrogels were mapped primarily to enzyme-responsive triggers and were most frequently applied in arthritis and intervertebral disk degeneration models.

Connections between multiple triggers and disease groups were observed for dual- or multi-responsive systems, indicating cross-category associations across heterogeneous pathological conditions.

Overall, the network representation demonstrates non-random associations between polymer families, trigger mechanisms, and disease models across the included studies.

## 4. Discussion

### 4.1. Integration of Outcomes

Rather than testing whether microenvironment-responsive polymers can function, the present analysis addresses a more practice-oriented question: Which material strategies have been repeatedly applied with reported benefit across diverse pathological settings? Because the majority of included studies reported improvement relative to untreated or baseline controls, a pattern that may partly reflect publication tendencies toward positive findings in preclinical research, the dataset represents a “solution landscape” of experimentally validated formulations rather than a balanced comparison between success and failure. This perspective enables identification of recurring design patterns linking polymer chemistry, trigger mechanism, and disease microenvironment.

Across disease models, therapeutic alignment appears structured rather than random. Outcome patterns stratified by polymer family correspond to characteristic functional groups and degradation pathways: ionizable carboxylates and amines in methacrylates, alginate, and chitosan support protonation–deprotonation equilibria and ionic swelling; ester hydrolysis governs PLGA behavior in vascular and tumor contexts; redox-active thioethers in PEG-PPS and labile coordination bonds in metal–organic frameworks enable ROS-sensitive destabilization; and glycosidic hyaluronic acid backbones are susceptible to enzyme-mediated cleavage in protease-rich tissues. These recurring motifs suggest physicochemical compatibility between polymer backbones and disease-associated microenvironmental features.

Importantly, trigger categories did not perform uniformly across contexts. Protonation equilibria and ion-exchange mechanisms showed the most consistent representation in colitis models, whereas ROS-responsive platforms predominated in tumor and atherosclerosis settings. Enzyme-responsive systems were concentrated in joint and degenerative models. Dual- or multi-responsive designs appeared across heterogeneous conditions and were frequently associated with robust therapeutic readouts, suggesting that combined activation mechanisms may buffer spatial and temporal variability within diseased tissues.

Viewed through a network framework, polymer–trigger–disease relationships form coherent clusters rather than diffuse associations. Polysaccharide- and methacrylate-based carriers converge on pH- and ion-sensitive strategies in inflammatory bowel disease; PEG–PPS systems and metal–organic frameworks cluster within oxidative tumor and vascular environments; hyaluronic-acid hydrogels map predominantly to enzyme-responsive joint disorders. Multi-node connections highlight the recurring use of composite materials in complex microenvironments.

Based on these patterns, Table 2 consolidates empirically recurrent polymer–trigger combinations by disease subtype together with their mechanistic rationale. Rather than prescribing a universally optimal material, this synthesis delineates a structured design space within which polymer selection can be aligned with dominant pathological conditions.

Taken together, the findings support the concept of mechanochemical compatibility as a central organizing principle in biodegradable polymer drug delivery. By integrating evidence across models, this work provides a formulation-oriented framework that moves beyond descriptive cataloging toward context-aware material selection.

### 4.2. Translational Relevance of Recommended Polymer-Trigger Systems

Table 2 synthesizes the most consistently reported polymer-trigger pairings across 65 preclinical studies, providing a formulation-oriented framework for disease-specific carrier selection. Rather than identifying universally superior materials, the data indicate that therapeutic performance is strongly influenced by the degree of mechanochemical compatibility between the carrier and the dominant pathological microenvironment.

In colitis and inflammatory bowel disease, polyanionic methacrylate copolymers (Eudragit) and polysaccharide matrices such as alginate and chitosan were frequently associated with reproducible outcomes. These systems exploit robust physicochemical constants of the intestinal milieu: protonation–deprotonation equilibria along the colonic pH gradient and dynamic ion-exchange processes. Carboxylate-bearing methacrylates undergo predictable dissolution transitions, while Ca^2+^-crosslinked alginate networks respond to Na^+^/Ca^2+^ exchange through formation and rearrangement of egg-box junction zones. Combined with chitosan-mediated mucoadhesion and ionic swelling, these mechanisms provide stable, scalable colon-targeted delivery platforms compatible with oral administration.

In arthritis and degenerative joint disorders, hyaluronic-acid-based hydrogels and HA-PEG composites were consistently associated with positive performance by leveraging matrix metalloproteinase (MMP) overexpression in inflamed synovial tissues. PEGylation improves retention and reduces nonspecific protein adsorption, whereas enzymatic cleavage of the HA backbone enables localized payload release. This trigger mechanism closely mirrors disease pathophysiology and supports the development of intra-articular depot formulations capable of sustained therapeutic exposure.

Atherosclerosis and vascular inflammation were dominated by ROS-responsive carriers, including PEG-PPS micelles and MOF- or nanozyme-based composites. Oxidation of thioether groups and destabilization of labile coordination bonds under oxidative stress can induce structural collapse and drug release. However, variability in local ROS concentrations suggests that adaptive or multi-responsive systems may be required for reliable human translation.

Solid tumors frequently employ dual pH+ROS-responsive or biomimetic composite systems. Their orthogonal activation pathways enable drug release even when one stimulus is heterogeneous or subthreshold, while nanoscale size and surface properties facilitate accumulation via the enhanced permeability and retention (EPR) effect. This redundancy may be particularly advantageous in spatially heterogeneous tumor microenvironments.

Collectively, these findings emphasize that polymeric drug-delivery systems tend to perform most consistently when chemical functionality, material physics, and disease-specific triggers are co-optimized. Translation is therefore unlikely to benefit from universal carrier platforms; instead, rational selection should prioritize alignment between ionizable groups, redox-sensitive motifs, or enzyme-labile structures and the dominant biochemical cues of the target pathology. Proteomics-derived epithelial biomarkers (e.g., TGM2, ICAM1, CEACAM1, ANXA1 [9]) further support the integration of disease-specific diagnostic profiling with targeted or trigger-matched formulation strategies in IBD.

### 4.3. Mechanistic Interpretation

Across stimuli-responsive platforms, efficacy patterns appear to closely track the intrinsic chemical reactivity of the materials within physiological ranges of pH, ionic strength, and redox potential. Polyanionic methacrylates exhibit predictable solubility shifts driven by protonation of pendant carboxyl groups, whereas polycationic polysaccharides such as chitosan rely on reversible amine protonation to induce swelling and mucoadhesion. Polyester carriers including PLGA and PLA undergo hydrolysis that is accelerated under acidic and inflammatory conditions, explaining enhanced release kinetics in tumors and damaged tissues.

Redox-responsive block copolymers such as PEG-PPS convert thioether oxidation into polarity changes that destabilize micellar cores, directly coupling drug release to oxidative stress gradients characteristic of atherosclerotic plaques and tumors. Hyaluronic-acid matrices exploit enzyme overexpression for site-specific degradation, while MOF- and nanozyme-based systems combine labile coordination bonds with catalytic modulation of reactive oxygen species, producing both therapeutic and environment-responsive effects.

From a translational perspective, these observations support the concept of mechanochemical matching, defined as the alignment between molecular reactivity and pathological milieu as a major determinant of in vivo performance. Systems activated by fundamental physicochemical properties of disease states, such as acidity, oxidative stress, or protease activity, tend to exhibit more reproducible outcomes. In contrast, platforms dependent on less stable or spatially variable stimuli may produce inconsistent results across models.

Importantly, each polymer family offers a defined activation pathway that can be tuned through composition, architecture, or crosslinking density. This design flexibility may enable the prediction of behavior under patient-specific biochemical conditions and provide a foundation for personalized formulation strategies. At a molecular level, responsiveness is governed by a limited set of recurring chemical motifs, including ionizable carboxylate and amine groups enabling pH- and ion-dependent behavior; hydrolysable ester backbones driving acid-accelerated degradation; redox-active thioethers mediating oxidative destabilization; enzyme-cleavable glycosidic networks; and labile coordination bonds in metal–organic frameworks. These motifs collectively define the physicochemical design space of biodegradable polymer carriers.

### 4.4. Regulatory and Translational Challenges

Considerable heterogeneity among the included studies, encompassing animal models, polymer architectures, dosing strategies, and outcome metrics, limits direct quantitative comparability. Incomplete reporting of sample sizes, variance measures, and release kinetics further constrains formal effect-size synthesis and necessitates a hybrid qualitative–quantitative approach.

Translational extrapolation is additionally complicated by differences between animal models and human pathological microenvironments. Although rodent models reproduce major disease hallmarks, electrolyte composition, pH distribution, oxidative status, and protease activity often differ from human tissues, potentially altering trigger responsiveness.

Future progress will require integration of mechanistic polymer design with experimental models that better capture patient-level biochemical variability. Biomarker-guided selection of responsive motifs, including protonatable groups, ion-sensitive crosslinks, redox-labile bonds, and enzyme-cleavable backbones, represents a promising strategy to align carrier behavior with disease-specific environments.

In summary, this analysis maps a coherent landscape of disease-linked polymer responsiveness but also highlights the need for standardized reporting, improved quantitative characterization, and translational models that reflect clinically relevant biochemical conditions.

### 4.5. Limitations of the Study

Despite providing a comprehensive synthesis of microenvironment-responsive polymer systems, this analysis has several limitations. Considerable heterogeneity among the included studies, in terms of animal models, polymer architectures, dosing regimens, administration routes, and outcome measures, limits direct quantitative comparability across experiments. In addition, incomplete reporting of sample sizes, variance measures, pharmacokinetic parameters, and release kinetics in some studies constrained formal meta-analytic effect size calculations and necessitated a hybrid qualitative–quantitative approach.

Translational extrapolation from animal models to human disease remains uncertain. Although rodent models reproduce key pathological features, differences in electrolyte composition, pH distribution, oxidative status, immune responses, and protease activity may alter trigger responsiveness in clinical settings. Furthermore, publication bias toward positive findings in preclinical research may lead to overrepresentation of successful formulations.

Finally, the analysis focused exclusively on biodegradable polymer systems published between 2020 and 2025 and indexed in major databases, which may exclude earlier foundational work or studies reported in other sources. Therefore, the framework proposed here should be interpreted as a contemporary evidence-based landscape rather than an exhaustive representation of all possible responsive materials.

## 5. Conclusions

Analysis of 65 preclinical in vivo studies identified non-random, disease-specific clustering of biodegradable polymer systems according to dominant activation mechanisms. Rather than being broadly interchangeable, polymer platforms tended to converge within distinct pathological contexts, indicating that disease microenvironments impose strong constraints on viable material designs.

Gastrointestinal inflammatory models were overwhelmingly associated with ionizable polysaccharide-based and methacrylate carriers operating through pH- and ion-dependent mechanisms, whereas enzyme-responsive hyaluronic acid systems were concentrated in joint and cartilage pathologies characterized by protease overexpression. In contrast, oxidative vascular disorders and solid tumors preferentially employed redox-responsive polyether systems and coordination-chemistry-driven platforms, reflecting the central role of reactive oxygen species in these conditions. Notably, composite and multi-responsive carriers were disproportionately represented in disease settings known for spatial and temporal heterogeneity, suggesting that robustness across fluctuating microenvironments may require orthogonal activation pathways.

These findings indicate that therapeutic performance is associated not simply with trigger compatibility but with the stability and dominance of specific biochemical cues within each disease. Pathologies with relatively uniform activation signals tend to favor single-mechanism systems, whereas complex or evolving microenvironments are more frequently addressed by hybrid architectures capable of maintaining functionality despite local variability.

By consolidating these recurrent associations into a polymer–trigger–disease framework, this study provides a practical basis for disease-calibrated formulation selection. Polymer choice may therefore be guided by the dominant microenvironmental drivers of the target pathology rather than by empirical material preference. Such an approach has direct implications for translational development, as alignment between carrier responsiveness and disease biology is likely to influence reproducibility and clinical viability.

Collectively, these findings suggest that, unlike platform-based pharmaceutical formulation, effective experimental drug-delivery systems exhibit disease-dependent material selection patterns. Incorporating pathological context into carrier choice may therefore support more rational and potentially personalized formulation strategies.

This framework may serve as a preliminary decision-support tool for selecting polymer carriers in personalized formulation development.

## Figures and Tables

**Table 1:** PICO framework applied in the present systematic review.

PICO Element	Description
Population (P)	Preclinical in vivo animal models and supplementary ex vivo/human serum studies in cancer and inflammatory conditions (e.g., colitis, hepatitis, atherosclerosis, sepsis, tumor models)
Intervention (I)	Ion- or pH-sensitive biodegradable polymer carriers (e.g., Eudragit, alginate, PLA/PLGA, pH-sensitive hydrogels, ROS-responsive polymers)
Comparator (C)	Non-sensitive polymer carriers/free drug/placebo control
Outcome (O)	Therapeutic efficacy (tumor size, inflammatory markers, survival), and the relationship between efficacy and host electrolyte milieu

**Table 2:** Recommended polymer-trigger systems by disease subtype, associated positive outcome rates, and mechanistic rationale.

Disease Subtype	Recommended Polymer Family/System	Dominant Trigger Mechanism	Evidence Strength *	Mechanistic Rationale
IBD/colitis	Eudragit (methacrylate copolymers)	pH-responsive	Strong	Enteric dissolution and protonation-deprotonation equilibria enable site-specific release in acidic inflamed intestinal regions
	Chitosan-based systems	pH/ionic	Moderate	Cationic swelling, mucoadhesion, and electrostatic interaction with negatively charged mucosa enhance retention and release
	PLGA formulations	pH-associated degradation	Supportive	Ester hydrolysis and autocatalytic acidification promote sustained release under inflammatory conditions
Arthritis/cartilage disorders	Hyaluronic acid hydrogels	Enzyme- responsive (MMPs, hyaluronidase)	Strong	Selective degradation in protease-rich synovial environment enables localized drug liberation
	Alginate or HA composites	ROS/enzyme	Moderate	Oxidative stress and enzymatic activity jointly promote matrix breakdown in inflamed joints
Atherosclerosis/cardiovascular disease	ROS-responsive polymers (e.g., PEG-PPS)	ROS	Strong	High oxidative burden in plaques activates redox- sensitive linkages, triggering payload release
	PEGylated nanoparticle systems	ROS/dual	Moderate	Prolonged circulation combined with oxidative activation enhances vascular targeting
Solid tumours	ROS-responsive systems	ROS	Strong	Elevated ROS flux in tumour microenvironment ensures efficient activation of redox- labile carriers
	Dual pH + ROS platforms	Combined stimuli	Moderate-strong	Concurrent acidity and oxidative stress provide robust activation despite spatial heterogeneity

* Evidence strength reflects the frequency of successful application across included studies rather than comparative therapeutic efficacy. “Positive outcome” denotes any statistically or biologically meaningful improvement in disease-related endpoints reported by the original authors relative to control conditions in the respective preclinical model. Across the included studies, authors reported improvement relative to control conditions; negative or null findings may be underrepresented due to publication and reporting biases.

## Data Availability

The original contributions presented in this study are included in the article/Supplementary Material. Further inquiries can be directed to the corresponding authors.

## Supplementary Materials

The following supporting information can be downloaded at: https://www.mdpi.com/article/10.3390/pharmaceutics18030330/s1. Table S1. Characteristics of the included studies (n = 65), including study identifier, disease model, polymer type, stimulus type (pH/ROS/ion/enzyme/dual), final stimulus classification, in vivo model, main therapeutic endpoint, and proposed mechanism of responsiveness. Ref. [28] has cited in Supplementary Materials.

## Abbreviations

The following abbreviations are used in this manuscript:

EPR	enhanced permeability and retention
HA	hyaluronic acid
IBD	inflammatory bowel disease
MMP	matrix metalloproteinase
MOF	metal–organic framework
PEG	poly(ethylene glycol)
PEG-PPS	poly(ethylene glycol)-block-poly(propylene sulfide)
PLA	poly(lactic acid)
PLGA	poly(lactic-co-glycolic acid)
ROS	reactive oxygen species
Tg	glass transition temperature
